# Sequence Variations in MYB (v-myb Myeloblastosis Viral Oncogene Homolog) Genes Impair Anthocyanin Biosynthesis and Contribute to Yellow Flower Phenotype in *Rehmannia glutinosa*

**DOI:** 10.3390/biom16010095

**Published:** 2026-01-07

**Authors:** Jianquan Tang, Qi Liu, Yuetong Liu, Hongyan Gao, Bing He, Ming Yue, Bin Li

**Affiliations:** 1 Shaanxi Engineering Research Centre for Conservation and Utilization of Botanical Resources, Xi’an Botanical Garden of Shaanxi Province, Institute of Botany of Shaanxi Province, Xi’an 710061, China; 2 College of Modern Agriculture and Biotechnology, Ankang University, Ankang 725000, China; 3 Baishui County Forestry Technology Extension Station, Weinan 715600, China; 4 Administration Office of Shaanxi Yellow River Wetland Provincial Nature Reserve, Weinan 714000, China; 5 Key Laboratory of Resource Biology and Biotechnology in Western China (Ministry of Education), College of Life Sciences, Northwest University, Xi’an 710069, China

**Keywords:** *Rehmannia glutinosa*, flower color variation, anthocyanins, MYB transcription factors, sequence variation

## Abstract

The corolla of *Rehmannia glutinosa* typically exhibits a stable reddish-purple color, but a naturally occurring yellow-flowered variant has recently been identified. To clarify the molecular basis of flower color variant, metabolomics, transcriptomics, and variant analyses were integrated. Metabolomic profiling revealed that the yellow phenotype was associated with lower anthocyanin levels and higher carotenoid levels. Specifically, the decreased cyanidin-3-O-glucoside led to a loss of red, while increased lutein provided the basis for the yellow color. Transcriptomic analysis revealed a downregulation of anthocyanin biosynthetic genes, including *CHS*, *CHI*, *F3H*, *DFR*, and *ANS*, in the yellow-flowered variant, and three S6-subgroup R2R3-MYB genes, including the known anthocyanin activator *RgMYB41* (*gene-DH2020_015992*), were downregulated. Variant analysis showed that A12S and G255E in the gene-DH2020_015992 transcription factor were predicted to markedly alter protein conformation and potentially impair regulatory function. Subcellular localization and transcriptional activation assays further supported the functional characterization of *gene-DH2020_015992* as a transcription factor. Collectively, these findings suggest that flower color variation in *R. glutinosa* is driven by MYB-mediated repression of anthocyanin biosynthesis and by carotenoid accumulation. This study provides a comprehensive genetic explanation for flower color variation in *R. glutinosa* and offers a theoretical foundation for floral pigmentation in plants.

## 1. Introduction

The diversity of flower coloration in plants is the result of long-term evolution shaped by natural selection. Flower color is not only a key feature of plant phenotype, but also the core object of evaluation and utilization of ornamental horticultural plants [1,2]. Flower coloration is closely linked to ecological adaptation, as many pollinators can selectively recognize and prefer specific colors, thereby enhancing plant reproductive success [3]. The formation of flower color is typically regulated by multiple factors, including pigment type and concentration, co-pigmentation effects, intracellular pH, and metal ions [2,4,5]. Among the three major classes of pigments known to contribute to flower coloration, anthocyanins and carotenoids are the primary determinants of flower color in most angiosperms, whereas betalains are restricted to a limited number of taxa [6,7]. Specifically, anthocyanins confer red to bluish-purple hues, while carotenoids generally impart yellow to orange tones [6]. Consequently, flower color diversity is not only a crucial research object in plant genetics, developmental biology, and evolutionary biology, but also holds significant value for horticultural breeding and the innovation of medicinal plant resources.

The anthocyanin biosynthetic pathway has been fully elucidated, and the key structural genes and their regulatory factors have been systematically identified [8,9]. Key enzymes in this pathway include chalcone synthase (*CHS*), chalcone isomerase (*CHI*), flavanone 3-hydroxylase (*F3H*), dihydroflavonol 4-reductase (*DFR*), and anthocyanidin synthase (*ANS*). The pathway of anthocyanin biosynthesis is also regulated by transcription factors such as MYB, bHLH, and WD40 proteins [10,11,12]. Within the plant R2R3-MYB gene family, the S6-subgroup is recognized as a key positive regulator of anthocyanin biosynthesis and has been validated in multiple species [10]. For instance, *AtMYB75*, *AtMYB90*, *AtMYB113*, and *AtMYB114* have been shown to directly activate *DFR* and *ANS* expression, thereby driving anthocyanin production in *Arabidopsis thaliana* [10,13]. In red-fleshed apple, the markedly upregulated *MdMYB10* acted as a core activator of anthocyanin accumulation, and its overexpression substantially increases pigment levels [14]. The transcription factor RcMYB3 bound to the promoters of *CHS* and *ANS*, activating their expression and promoting anthocyanin accumulation in *Rehmannia chingii*. Functional studies through both overexpression and targeted knockout of *RcMYB3* confirmed its critical role as a regulator of anthocyanin biosynthesis [15]. Moreover, microRNAs (miRNAs) also contribute to anthocyanin regulation primarily by targeting transcription factors rather than structural genes, indirectly influencing pigment production. For example, miR858 suppressed anthocyanin synthesis by repressing specific MYB genes, whereas miR156 alleviated repression on anthocyanin biosynthesis by targeting SPL genes [16,17].

*Rehmannia glutinosa* is an important Chinese herbal medicine plant, long utilized in traditional Chinese medicine. As a historically cultivated medicinal herb, it exhibits stable morphological traits, with tubular reddish-purple corollas serving as a key taxonomic feature. Despite its medicinal significance, studies on the molecular mechanisms of flower color formation remain limited in *R. glutinosa*. A total of 58 candidate genes involved in anthocyanin biosynthesis were identified, including *RpF3H2*, *RpDFR2*, *RpANS1*, *RpANS2*, and the S6-subgroup R2R3-MYB *RpMYB1* in *Rehmannia piasezkii*. Overexpression of *RpMYB1* activated downstream structural genes of the anthocyanin pathway and enhanced anthocyanin accumulation in tobacco leaves [18]. Overexpression of *RcMYB1* and *RcMYB3* significantly increased anthocyanin content in leaves and tuberous roots in *R. chingii*, particularly in *RcMYB3* transgenic lines, which exhibited substantial elevation in total anthocyanins and cyanidin-3-O-glucoside levels. Conversely, CRISPR/Cas9-mediated knockout of *RcMYB3* led to white-flowered phenotypes accompanied by a marked reduction in anthocyanin content [15]. Previous studies have suggested that MYB transcription factors play a central role in regulating anthocyanin biosynthesis and flower color formation in *Rehmannia* species. Despite the generation of flower-color variants in *Rehmannia* via modern biotechnology under controlled conditions, naturally occurring variants with stable inheritance are exceedingly scarce. Recently, a stable, heritable yellow-flowered variant of *R. glutinosa*, phenotypically distinct from the typical reddish-purple corolla, was found in a naturally occurring population. This natural variation expands the genetic diversity of *R. glutinosa*, and elucidating its metabolic basis and molecular mechanism will provide deeper insights into flower color variation in plants.

Although numerous studies have elucidated the regulatory roles of MYB transcription factors in anthocyanin biosynthesis through transgenic or genome-editing approaches, it remains largely unexplored whether stably inherited sequence variations in MYB genes occur in natural populations and whether such variations are associated with flower color phenotypic differences. Based on a stably inherited yellow-flowered *R. glutinosa* variant identified in a natural population, we proposed the hypothesis that nonsynonymous mutations in key MYB transcription factors may be associated with suppressed anthocyanin biosynthesis and flower color variation by affecting protein structural stability and transcriptional regulatory capacity. Guided by this hypothesis, we integrated widely targeted metabolomics, transcriptomic analysis, whole-genome resequencing, and molecular biology assays to systematically characterize the metabolic features, transcriptional changes, and potential genetic variation underlying flower color variation in yellow-flowered *R. glutinosa*. This study aimed to provide new insights into the genetic basis of flower color variation in *R. glutinosa* and to offer a theoretical reference for studies on floral pigmentation and diversity in plants.

## 2. Materials and Methods

### 2.1. Plant Materials

The *R. glutinosa* materials used in the present study were collected from a natural population in Baishui County, Weinan City, Shaanxi Province, China. The sampled plants included wild-type individuals with the typical reddish-purple corolla (RH) and a naturally occurring yellow-flowered variant with stable heredity (YH). For each type, tissues were collected from at least ten healthy plants. During sampling, the natural habitat and external morphological characteristics of both RH and YH plants were recorded, with particular attention to floral traits, and the corollas were photographed in the field. The corollas were longitudinally dissected to document internal structural and morphological details. Freshly collected corollas and leaves were immediately flash-frozen in liquid nitrogen and transported to the laboratory, where they were stored at −80 °C. The corolla tissues of each group were thoroughly homogenized and randomly divided into three portions: one for metabolomic analysis, one for whole-transcriptome sequencing, and one for total RNA extraction. Leaf tissues from YH plants were used for whole-genome resequencing.

### 2.2. Widely Targeted Metabolomic Analysis

Metabolomic profiling was performed on corolla tissues of RH and YH plants, with three biological replicates per group. The metabolomic analysis was outsourced to Biomarker Technologies (Beijing, China). Corolla samples were vacuum freeze-dried, after which 50 mg of each sample was weighed into a centrifuge tube and extracted with 1000 μL of extraction solvent (methanol: acetonitrile: water = 1:2:1, *v*/*v*/*v*). The mixture was vortexed for 30 s and homogenized with steel beads using a tissue grinder at 45 Hz for 10 min, followed by ultrasonication in an ice-water bath for 10 min to ensure thorough metabolite extraction. The samples were then placed at −20 °C for 1 h and centrifuged at 12,000 rpm for 15 min at 4 °C. A total of 300 μL of the supernatant was filtered through a 0.22 μm organic membrane and transferred to injection vials. To monitor instrumental stability, 10 μL from each sample was pooled to prepare a quality control (QC) sample. Metabolite detection was conducted using a UPLC-ESI-MS/MS platform, in which the UPLC system was Waters Acquity I-Class PLUS and the mass spectrometer was Applied Biosystems QTRAP 6500+. Raw peak area data were normalized by the total peak area, followed by principal component analysis (PCA) and Spearman correlation analysis to evaluate the stability and consistency of QC samples and biological replicates. Metabolite identification and classification were performed with reference to the KEGG, HMDB, and LipidMaps databases [19,20,21]. Differentially accumulated metabolites (DAMs) were screened based on variable importance in projection (VIP) values obtained from OPLS-DA, fold change (FC), and statistical significance. The thresholds were set as FC > 1, *p* < 0.05, and VIP > 1. KEGG pathway enrichment analysis was conducted using the OmicShare platform [22].

### 2.3. RNA-Seq Sequencing and Data Analysis

Transcriptome analysis was performed on corolla tissues of RH and YH plants, with three biological replicates per group. RNA-seq was conducted by Biomarker Technologies (Beijing, China). Total RNA was extracted using the RNAprep Pure Plant Kit (Tiangen, Beijing, China). For each sample, 1 μg of high-quality RNA was used to construct sequencing libraries with the Hieff NGS Ultima Dual-mode mRNA Library Prep Kit for Illumina (Yeasen Biotechnology, Shanghai, China). After library quality assessment using an Agilent 2100 Bioanalyzer, paired-end sequencing (150 bp) was carried out on the Illumina NovaSeq platform. Raw sequencing reads were subjected to quality control, including adaptor removal and filtering of low-quality sequences. Clean reads were aligned to the reference genome using HISAT2 [23]. The reference genome sequence was obtained from NCBI, with GenBank accession KX636157.1 and RefSeq accession NC_034308.1. Transcript assembly and the identification of known and novel transcripts were performed using StringTie (v2.2.3) [24]. Functional annotation of transcripts was conducted against the NR, Swiss-Prot, GO, COG, KEGG, KOG, and Pfam databases [25,26,27,28,29]. Gene expression levels were quantified as FPKM (Fragments Per Kilobase Million) [30]. Differential expression analysis was performed using DESeq2, with differentially expressed genes (DEGs) defined as those with an adjusted *p*-value (FDR) < 0.01 and a fold change ≥ 2 [31]. The raw RNA-seq data generated in the present study have been deposited in the Genome Sequence Archive (GSA) at the China National Center for Bioinformation (CNCB) under the accession number CRA032666.

### 2.4. Whole-Genome Resequencing-Based Variant Analysis

Whole-genome resequencing was conducted to identify genome-wide sequence variations in yellow-flowered *R. glutinosa*, including SNPs and InDels, by mapping reads to the reference genome (GenBank: KX636157.1; RefSeq: NC_034308.1). Genomic DNA was extracted from YH leaf tissues using a modified CTAB method. DNA sequencing libraries with an average insert size of 350 bp were constructed and sequenced on the Illumina NovaSeq platform to generate 150 bp paired-end reads. Raw sequencing data were subjected to quality control and filtering using *fastp* to remove adaptor contamination and low-quality reads, yielding high-quality clean data [32]. Clean reads were aligned to the reference genome using BWA-MEM2 [33]. The alignment files were subsequently sorted and deduplicated with SAMtools [34]. Variant calling was performed using GATK, including initial identification, quality recalibration, and filtering of SNPs and InDels, ultimately generating a high-confidence variant dataset [35]. The genomic distribution and functional effects of the mutation were analyzed using SnpEff (v4.3.) [36]. The raw data from whole-genome resequencing in the present study were deposited in the GSA database of CNCB under accession number CRA032741.

### 2.5. Phylogenetic Analysis

Multiple sequence alignments were performed using the MAFFT algorithm [37]. An unrooted neighbor-joining (NJ) tree was inferred from the amino-acid alignments. Evolutionary distances were computed using the *p*-distance model under the assumption of uniform substitution rates among sites. Branch support was evaluated by 1000 bootstrap replicates. Gaps and missing data were treated by partial deletion, with a 95% site coverage cutoff. Phylogenetic trees were constructed in MEGA X (v10.2.6) [38], and tree visualization was conducted with Evolview (v3.0) [39]. Classification of R2R3-MYB genes in *R. glutinosa* was performed following the subgrouping criteria established for the *Arabidopsis* R2R3-MYB family [13].

### 2.6. Protein Structural Modeling and Conformational Analysis

The three-dimensional structural models of target proteins were predicted using the AlphaFold Server (https://alphafoldserver.com/, accessed on 15 October 2025). Protein visualization and structural analyses were performed in PyMOL (v2.5.0), and structural alignment was conducted using the *align* command. The global root mean square deviation (RMSD) was calculated to evaluate the impact of amino acid substitutions on the overall protein conformation. To assess local structural perturbations introduced by mutations, a local peptide segment comprising five upstream and five downstream amino acid residues flanking each mutation site was extracted, and the local RMSD was computed. Changes in distances (*ΔD*) between the Cα atom of the mutated residue and its neighboring residues, defined as residues within 8 Å of the mutation-site Cα, were calculated using the following formula:

(1) $$\Delta D=D_{MUT}-D_{WT}$$

where $D_{MUT}$ and $D_{WT}$ represent the interatomic distances between corresponding atoms before and after mutation, respectively.

### 2.7. Subcellular Localization Analysis

Subcellular localization was examined using a transient expression system in *Nicotiana benthamiana* leaves following previously described procedures [40,41]. The coding sequences (CDS) of target genes were cloned into the *pCAMBIA1305-GFP* vector, and recombinant constructs were introduced into *Agrobacterium tumefaciens* strain GV3101 for agroinfiltration of tobacco leaves [42]. The green fluorescence signal in tobacco leaf cells was observed using a laser scanning confocal microscope (Leica, Mannheim, Germany). Primers used for the subcellular localization assay are listed in Table S1.

### 2.8. Transcriptional Activation Assay

The assay of transcriptional activation activity was performed using a yeast expression system following previously described methods [42]. The *pGBKT7* vector and the *Saccharomyces cerevisiae* strain AH109 were employed for the assay. Yeast cell expressing foreign genes was grown on the medium lacking tryptophan or tryptophan and histidine to detect the transcriptional activation activity. Primers used for the transcriptional activation assay are listed in Table S1.

### 2.9. Statistical Analysis

Statistical analyses were performed using R software (v4.5.1). Differences between groups were assessed using two-tailed independent-sample *t*-tests. Significance levels were defined as follows: ns (not significant), *p* ≥ 0.05; *p* < 0.05 (*)*; p <* 0.01 (**); *p <* 0.001 (***). Correlation and linear regression analyses were conducted using Python (v3.11.7). Data visualization was performed using GraphPad Prism 8.0.

## 3. Results

### 3.1. Comparison of Color Differences Between Two R. glutinosa Materials

**Figure 1:**
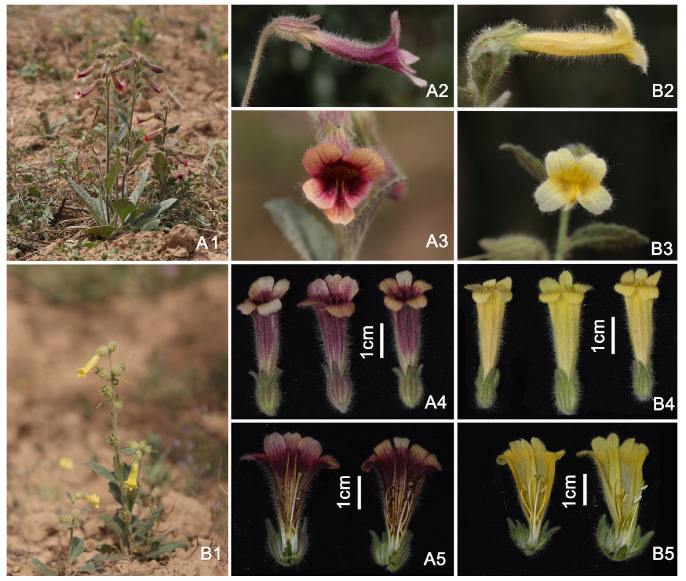
Morphological comparison between wild-type and the yellow-flowered materials in *R. glutinosa.* (**A1**–**A5**) Wild-type material; (**B1**–**B5**) Yellow-flowered mutant. (**A1**,**B1**) Habitat of the plants; (**A2**,**B2**) Lateral view of the flowers; (**A3**,**B3**) Front view of the flowers; (**A4**,**B4**) Lateral view of the detached corolla; (**A5**,**B5**) Longitudinal section of the corolla.

A comparative morphological analysis between wild-type and the yellow-flowered variant revealed that the two types differed markedly only in flower color, while no discernible differences were observed in other morphological traits (Figure 1). Both phenotypes were found growing in the same natural habitat. The wild-type plants exhibited a typical deep reddish-purple corolla, whereas the yellow-flowered mutant displayed a uniformly bright yellow corolla, forming a striking contrast (Figure 1). Lateral views of excised corollas further demonstrated that wild-type flowers possessed prominent dark purple longitudinal stripes on the abaxial side of the corolla tube, whereas these stripes were completely absent in the yellow-flowered type (Figure 1). The conspicuous divergence in floral coloration indicated potential alterations in anthocyanin biosynthesis, carotenoid biosynthesis, or transcriptional regulation. Subsequent integrative multi-omics analyses were therefore expected to elucidate the molecular mechanisms underlying the yellow corolla phenotype in *R. glutinosa*.

### 3.2. Reduced Anthocyanin Levels and Enhanced Carotenoid Accumulation in Yellow Corolla Formation

**Figure 2:**
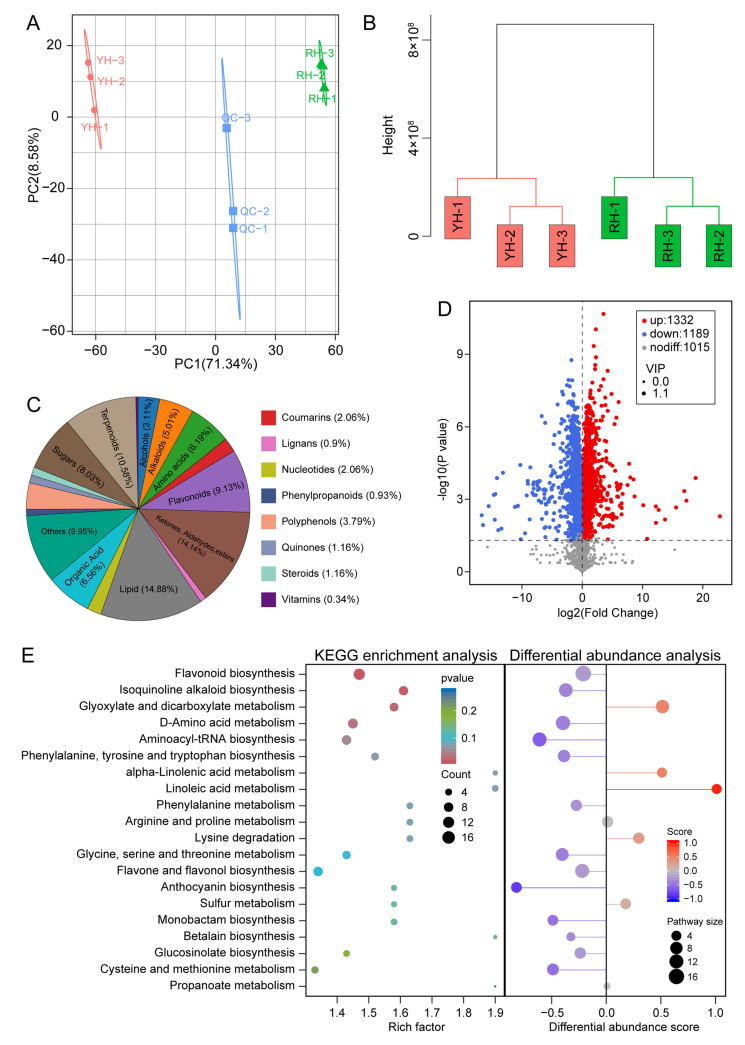
Metabolomic analysis of corollas in *R. glutinosa*. (**A**) PCA analysis of metabolomic data. (**B**) Cluster analysis of metabolomic data. (**C**) Classification of metabolites. (**D**) Differential analysis of metabolites. (**E**) KEGG enrichment analysis of differentially accumulated metabolites.

To elucidate the metabolic basis underlying the floral color differences between RH and YH, a widely targeted metabolomic analysis was performed on corolla tissues from both phenotypes. The PCA result showed a clear separation between RH and YH samples along PC1, indicating substantial metabolic divergence between the two groups (Figure 2A). Hierarchical clustering further revealed that biological replicates clustered tightly within each group, confirming high intra-group consistency (Figure 2B). A total of 3536 metabolites were detected and classified into 18 structural categories, with lipids representing the most abundant group (Figure 2C). To identify key metabolites accompanied by the color variation, differential metabolite analysis was conducted. The OPLS-DA model effectively distinguished the metabolic profiles of RH and YH, and permutation tests verified the model’s robustness and lack of overfitting (Figure S1A,B). Using RH as the reference, a total of 2521 DAMs were identified in YH, among which 1332 were up-regulated, and 1015 were down-regulated (Figure 2D). KEGG enrichment analysis revealed that these DAMs were significantly associated with several metabolic pathways, including carotenoid biosynthesis, flavonoid biosynthesis, and anthocyanin biosynthesis (Figure 2E).

**Figure 3:**
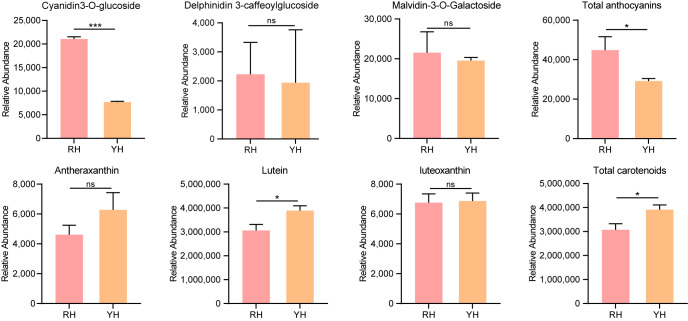
Metabolic profiles of anthocyanins and carotenoids in *R. glutinousa*. Data are presented as mean ± SD. Group differences were evaluated using a two-tailed independent-sample *t*-test: ns (not significant), *p* ≥ 0.05; *: *p* < 0.05; ***: *p* < 0.001.

Considering the enrichment of carotenoid biosynthesis and anthocyanin biosynthesis pathways, the specific metabolic spectra of anthocyanins and carotenoids in RH and YH were further analyzed, and three anthocyanins and three carotenoids were identified. Compared with RH, cyanoidin-3-O-glucoside, delphinidin 3-caffeoylglucoside, and malvidin-3-O-galactoside in YH decreased, among which cyanoidin-3-O-glucoside decreased significantly (Figure 3). In contrast, two carotenoids, antheraxanthin and lutein, accumulated in YH, and lutein displayed a significant increase (Figure 3). Taken together, the total anthocyanin content in YH corolla decreased while the total carotenoid content increased. This shift in pigment composition likely represents the key metabolic basis underlying the yellow corolla phenotype in *R. glutinosa*.

### 3.3. Suppression of the Anthocyanin Biosynthetic Pathway in Yellow-Flowered R. glutinosa

**Figure 4:**
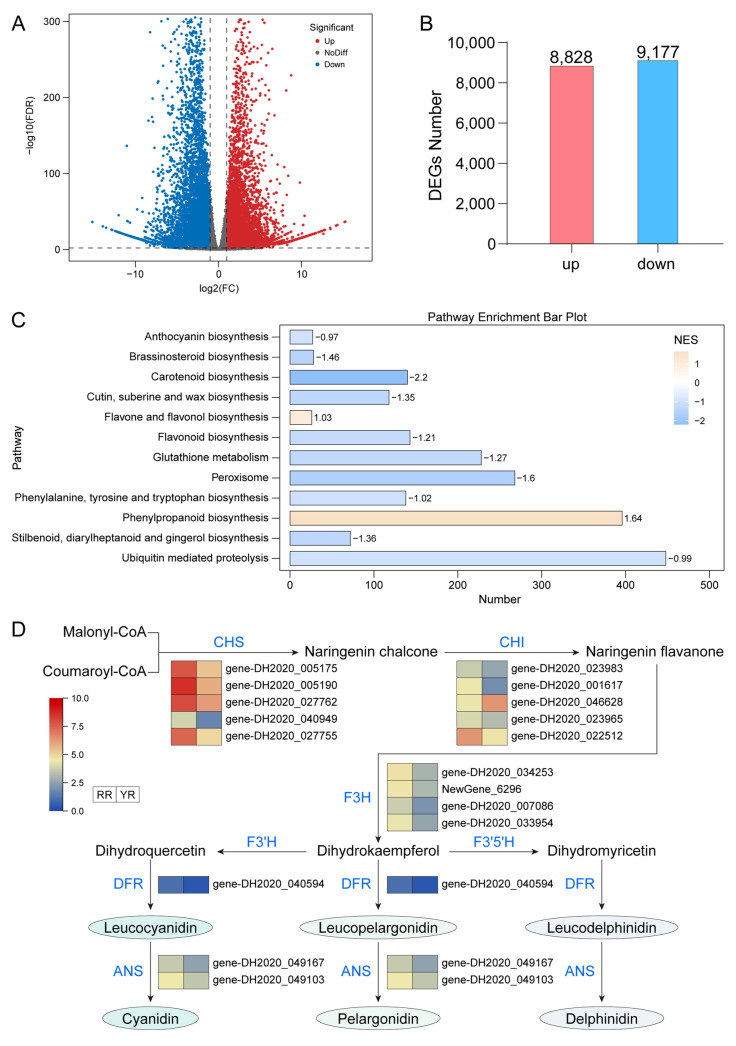
Transcriptomic analysis of floral tissues in *R. glutinousa*. (**A**) Differential gene expression analysis. The two vertical dashed lines correspond to log₂(FC) = 1 and log₂(FC) = –1, respectively; the horizontal dashed line indicates –log₁₀(FDR) = 2. (**B**) Statistics of the number of differentially expressed genes. (**C**) GSEA enrichment analysis of differentially expressed genes. (**D**) Expression patterns of key enzyme genes involved in the anthocyanin biosynthesis pathway in *R. glutinousa*.

To elucidate the transcriptional regulatory mechanisms underlying the formation of the yellow corolla, RNA-seq analysis was performed. The sequencing results showed that all samples exhibited Q30 values exceeding 98%, and more than 90% of high-quality clean reads were successfully mapped to the reference genome (Tables S2 and S3). Both hierarchical clustering and PCA indicated strong reproducibility among biological replicates, confirming the high quality of the transcriptomic data and their suitability for downstream expression analyses (Figure S2A,B). Differential expression analysis identified a total of 18,005 DEGs, including 8828 upregulated and 9177 downregulated genes (Figure 4A,B). Gene Set Enrichment Analysis (GSEA) revealed that these DEGs were associated with multiple metabolic pathways, such as anthocyanin biosynthesis, carotenoid biosynthesis, flavone and flavonol biosynthesis, and flavonoid biosynthesis (Figure 4C). Given the metabolomic evidence indicating reduced anthocyanin accumulation in YH, we focused on genes involved in the anthocyanin biosynthetic pathway. Compared with RH, YH corollas exhibited pronounced downregulation of key structural genes in this pathway, including *CHS*, *CHI*, *F3H*, *F3′H*, *F3′5′H*, *DFR*, and *ANS* (Figure 4D). These results indicated that the anthocyanin synthesis pathway was inhibited in YH, which likely represented a molecular basis for the emergence of the yellow corolla phenotype.

### 3.4. Downregulation of S6-Subgroup R2R3-MYB Genes in Yellow-Flowered R. glutinosa

**Figure 5:**
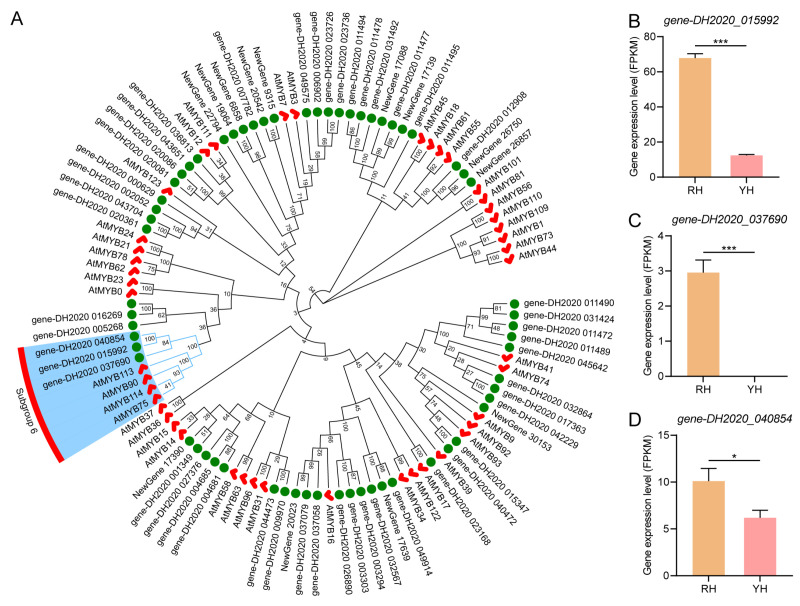
Phylogenetic analysis and gene expression profiling of S6-subgroup R2R3-MYB genes in *R. glutinosa*. Details of S6-subgroup R2R3-MYB proteins can be found in Table S4. (**A**) Phylogenetic analysis of R2R3-MYB genes in *R. glutinosa*. Red checkmarks indicated MYB proteins from *A. thaliana*. Green solid circles indicated MYB proteins from *R. glutinosa*. Blue background represented MYB members belonging to Subgroup S6. (**B**) Expression level of *gene-DH2020_015992*. (**C**) Expression level of *gene-DH2020_037690*. (**D**) Expression level of *gene-DH2020_040854*. Group differences were evaluated using a two-tailed independent-sample *t*-test. *: *p* < 0.05; ***: *p* < 0.001.

To further investigate the regulatory factors associated with the anthocyanin synthesis pathway in YH, S6-subgroup R2R3-MYB, an important transcription factor regulating the anthocyanin synthesis pathway, was analyzed in *R. glutinosa*. Phylogenetic analysis revealed that *gene-DH2020_040854*, *gene-DH2020_037690*, and *gene-DH2020_015992*, were clustered in a branch with the S6-subgroup R2R3-MYB, AtMYB75, AtMYB90, AtMYB113, and AtMYB114 in *A. thaliana*, indicating that these proteins belong to the S6-subgroup in *R. glutinosa* (Figure 5A). Transcriptomic analysis showed that the expression levels of all three MYB genes were significantly reduced in YH compared with the wild type (Figure 5B–D). Meanwhile, multiple structural genes involved in the anthocyanin biosynthetic pathway were also strongly downregulated in YH. Taken together, these results indicated that the reduced expression of S6-subgroup MYB genes was closely associated with the suppressed transcription of anthocyanin biosynthetic genes in yellow-flowered *R. glutinosa*.

### 3.5. Identification and Distribution of SNP/InDel Variants in Yellow-Flowered R. glutinosa

**Figure 6:**
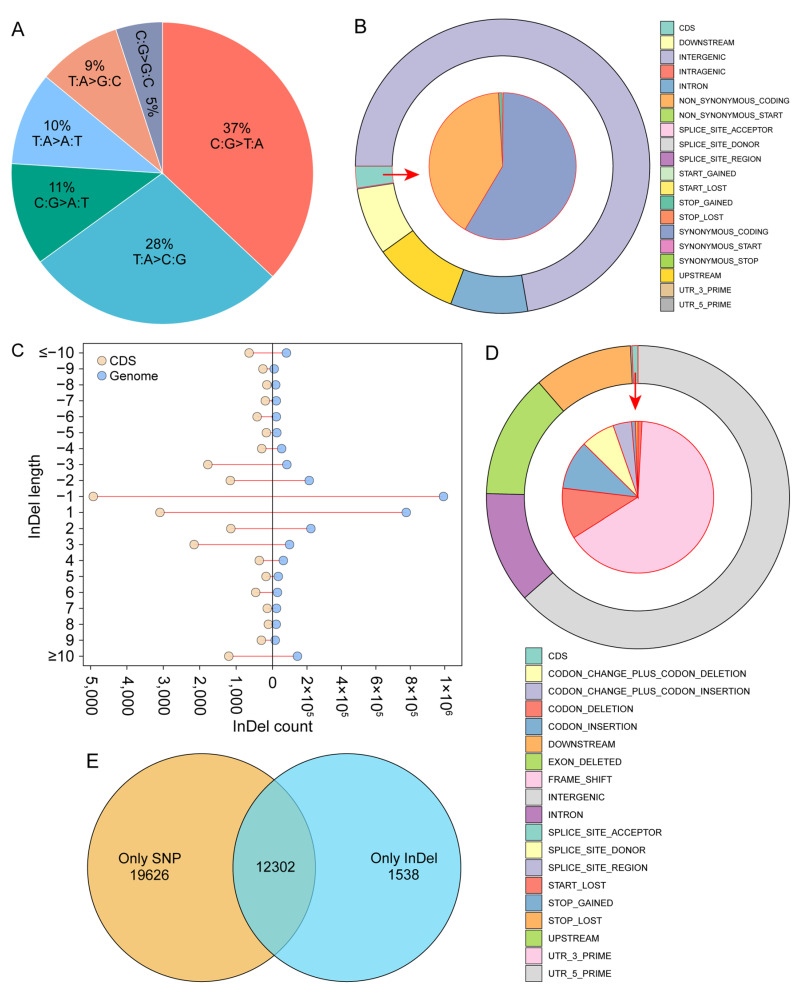
Whole-genome resequencing–based variant analysis of yellow-flowered in *R. glutinosa*. (**A**) Classification of SNP types. (**B**) Distribution of SNPs across different genic regions. The inner ring pointed to by the red arrows indicates the distribution of mutations on the CDS. (**C**) Length distribution of InDels in the whole genome and CDS. (**D**) Distribution of InDels across different genic regions. The inner ring pointed to by the red arrows indicates the distribution of mutations on the CDS. (**E**) Genes containing nonsynonymous SNPs and InDels occurring within CDS regions.

To investigate genomic differences between the two phenotypes, whole-genome resequencing–based variant analysis was performed for the yellow-flowered variant. The resequencing data exhibited excellent quality, with a Q30 score of 98.65%, a mapped read ratio of 98.99%, and an average sequencing depth of 24×, indicating that the dataset was reliable and suitable for subsequent analyses of genetic variation and functional annotation (Table S5). The distributions of SNP depth and neighboring SNP distance further supported the high confidence of SNP detection across the genome (Figure S3A,B). A total of 12,857,485 SNPs were identified, which were categorized into six mutation types, among which C:G > T:A and T:A > C:G transitions were the most abundant (Table S6, Figure 6A). SNP annotation revealed that approximately 72.3% of all SNPs were located in intergenic regions (Figure 6B). A total of 302,698 SNPs were found in CDS, including 178,115 synonymous and 123,451 non-synonymous substitutions (Figure 6B). The length distribution of InDel together with their depth distribution supported the reliability of InDel identification across the genome (Figure S3C,D). Approximately 1.7 million InDels were detected, the vast majority of which were located in non-coding regions, whereas only 0.65% occurred within CDS regions. Multiple types of InDels were identified in CDS regions, with ±1 bp and ±2 bp mutations being the most common. Frameshift mutation (FRAME_SHIFT) was the largest, with 12,520 (Table S7, Figure 6C,D). To identify the genes whose mutation may lead to functional changes, the genes with non-synonymous SNPs and InDels in the CDS region were screened. The results showed that there were 31,928 non-synonymous mutation SNP genes, 13,840 InDel genes, and 12,302 overlapping genes (Figure 6E).

**Figure 7:**
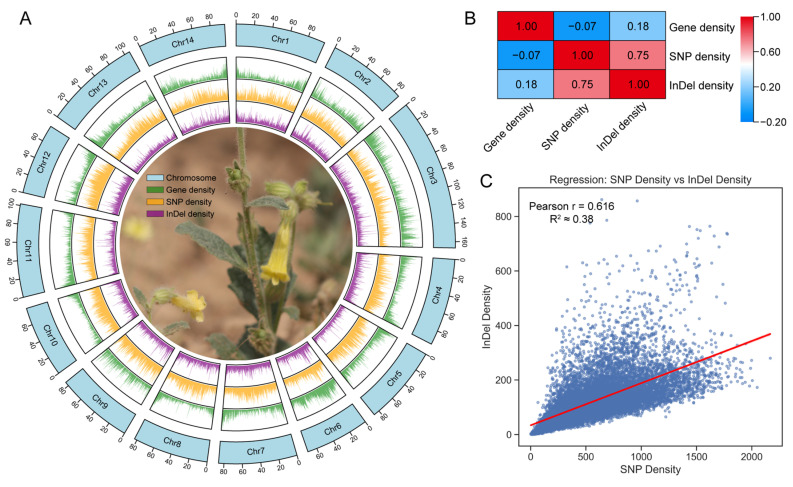
(**A**) Chromosomal distribution of SNPs and InDels in *R. glutinosa*. (**B**) Correlation analysis among gene density, SNP density, and InDel density. (**C**) Regression analysis between SNP density and InDel density.

To further investigate the distribution characteristics of mutation across the genome, gene density, SNP density, and InDel density were visualized on chromosomes, followed by correlation analyses (Figure 7A). The correlation analysis revealed that gene density showed almost no correlation with SNP density (Spearman’s r = −0.073) and only a weak correlation with InDel density (Spearman’s r = 0.182), indicating that the occurrence of mutation was largely uncoupled from gene density (Figure 7B). In contrast, SNP density and InDel density exhibited a significant positive correlation (Spearman’s r = 0.75, Pearson’s r = 0.616, R^2^ ≈ 0.38), suggesting that SNP and InDel may arise under similar mutational backgrounds or be driven by shared genomic processes (Figure 7B,C).

### 3.6. Nonsynonymous Variants in S6-Subgroup R2R3-MYB Genes in R. glutinosa

**Figure 8:**
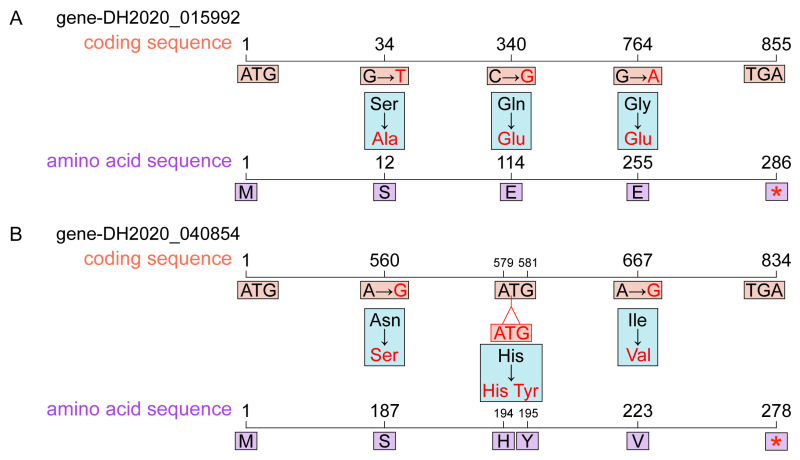
Nonsynonymous SNPs and InDels in the CDS regions of MYB genes in *R. glutinosa*. (**A**) Nonsynonymous SNPs in the CDS region of *gene-DH2020_015992*. (**B**) Nonsynonymous SNPs and InDels in the CDS region of *gene-DH2020_040854*. The asterisk (*) indicated the position corresponding to the stop codon in the protein translation.

To further elucidate the molecular basis underlying the downregulation of the S6-subgroup R2R3-MYB genes (*gene-DH2020_040854*, *gene-DH2020_037690*, and *gene-DH2020_015992*) in YH, nonsynonymous SNPs and InDels within their CDS were examined. No nonsynonymous SNPs or InDels were detected in the CDS of *gene-DH2020_037690*. Three nonsynonymous SNPs were found in the CDS of *gene-DH2020_015992*: G-to-T substitution at position 34, C-to-G substitution at position 340, and G-to-A substitution at position 764. These mutations resulted in amino acid changes: Serine to Alanine at position 12, Glutamine to Glutamic acid at position 114, and Glycine to Glutamic acid at position 255 (Figure 8A). Two nonsynonymous SNPs and one three-base insertion were identified in the CDS of *gene-DH2020_040854*: A-to-G substitution at position 560, A-to-G substitution at position 667, and ATG insertion between positions 579 and 580. These mutations resulted in amino acid changes: Asparagine to Serine at position 187, Isoleucine to Valine at position 223, and the insertion of an additional Tyrosine residue between positions 194 and 195 (Figure 8B).

### 3.7. Structural Alterations in Gene-DH2020_040854 and Gene-DH2020_015992

**Figure 9:**
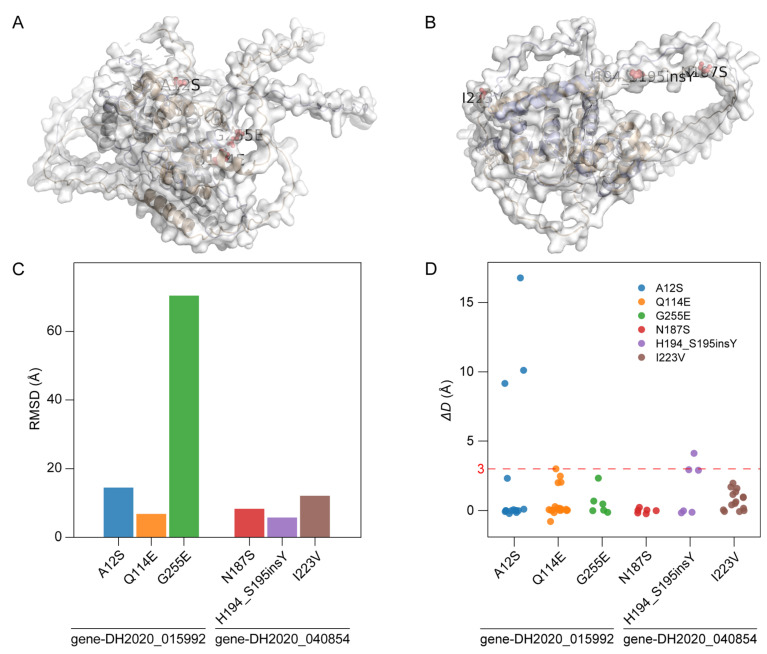
Molecular modeling and structural comparison of MYB proteins in *R. glutinosa*. (**A**) Molecular modeling and structural comparison between the wild-type and mutant *gene-DH2020_015992* proteins. (**B**) Molecular modeling and structural comparison between the wild-type and mutant *gene-DH2020_040854* proteins. Light blue: Wild-type protein structure. Gold: Mutant protein structure. Red spheres: Mutation sites on the mutant protein. (**C**) RMSD analysis of the variant sites. (**D**) Analysis of distance changes between variant sites and their neighboring residues. The red dotted line indicates where *ΔD* equals 3 Å.

To evaluate the effects of nonsynonymous mutations on protein spatial conformation, molecular modeling and structural alignment analyses were performed for both the wild-type and mutant proteins of *gene-DH2020_040854* and *gene-DH2020_015992* (Figure 9A,B). The global RMSD between the mutant and wild-type proteins of *gene-DH2020_015992* reached 13.739 Å, which was markedly higher than that of *gene-DH2020_040854* (6.601 Å), indicating that *gene-DH2020_015992* underwent more substantial overall conformational deviations (Table S8). Local structural analyses revealed that the A12S, Q114E, and G255E mutations in *gene-DH2020_015992* exhibited local RMSD values of 14.482 Å, 6.819 Å, and 70.459 Å, respectively, with the G255E mutation showing an exceptionally high local RMSD (Figure 9C, Table S9). In contrast, the N187S, H194_S195insY, and I223V mutations in *gene-DH2020_040854* displayed relatively modest structural perturbations, with local RMSD values of 8.314 Å, 5.754 Å, and 12.117 Å, respectively, suggesting that structural changes in *gene-DH2020_040854* were comparatively mild (Figure 9C, Table S9). The analysis of distance changes between Cα atoms and neighboring residues revealed that most *ΔD* values were below 1.00 Å, but several residues exhibited pronounced deviations (Figure 9D, Table S10). The A12S mutation in *gene-DH2020_015992* had the most significant structural impact, with *ΔD* values of 16.78 Å, 10.11 Å, and 9.16 Å for neighboring residues 262, 265, and 266, respectively. In comparison, *gene-DH2020_040854* showed a notable ΔD only between the H194_S195insY mutation and the adjacent residue 198 (4.12 Å). Although the G255E mutation displayed a maximum *ΔD* of only 2.3 Å, its exceptionally high local RMSD suggested instability in the surrounding structural region (Figure 9D, Table S10). Collectively, these findings indicate that the nonsynonymous mutations in *gene-DH2020_015992*, particularly A12S and G255E, may induce more severe global and local structural perturbations, potentially impairing the functional performance of this transcription factor.

### 3.8. Molecular Characterization of the Gene-DH2020_015992 Protein

**Figure 10:**
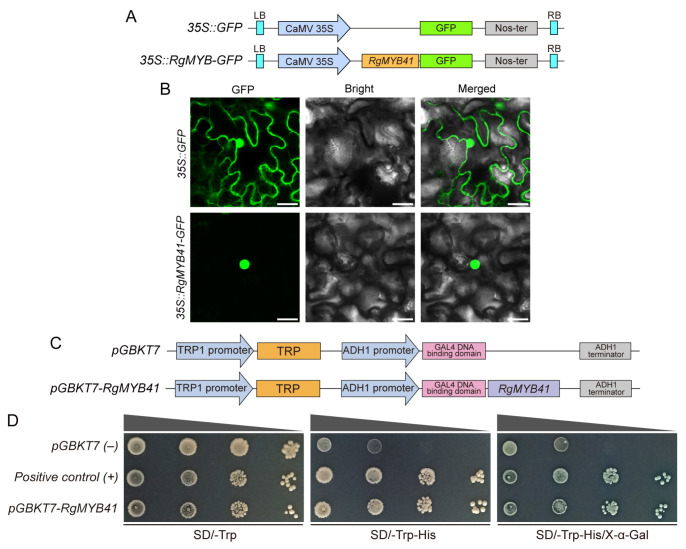
Subcellular localization and transcriptional activation analysis of the *gene-DH2020_015992* protein. (**A**) Schematic representation of the *35S::GFP* and *35S::RgMYB41-GFP* constructs used in the subcellular localization experiment. (**B**) Fluorescence signals of GFP protein and RgMYB41-GFP fusion protein in tobacco leaf cells were observed under a confocal laser scanning microscope, with a scale bar of 25 µm. (**C**) Schematic representation of the *pGBKT7* and *pGBKT7-RgMYB41* vectors used in the transcriptional activation assay. (**D**) Representative images of the results from the transcriptional activation assay, with *pGBKT7* serving as the negative control.

To further elucidate the molecular characteristics of the *gene-DH2020_015992* transcription factor, subcellular localization and transcriptional activation assays were performed. According to a previous report, this transcription factor has been designated RgMYB41, and this name was adopted in the present study. Subcellular localization analysis revealed that the GFP fluorescence of the RgMYB41–GFP fusion protein was exclusively detected in the nuclei of tobacco leaf epidermal cells under transient expression conditions, with no detectable fluorescence in the cytoplasm (Figure 10A,B). This result suggested that RgMYB41 possesses nuclear localization potential, which is consistent with its predicted role as a transcription factor. The yeast assay revealed that yeast cells expressing *RgMYB41* could grow normally on medium lacking tryptophan and histidine. Moreover, these transformed yeast cells exhibited a pale blue coloration on double-deficient medium supplemented with X-α-Gal, confirming that RgMYB41 possessed transcriptional activation activity (Figure 10C,D).

## 4. Discussion

Flower color variation is a widespread phenomenon in nature, playing an important role in providing valuable genetic resources for ornamental trait improvement. Traditionally, a highly stable reddish-purple corolla is observed in *R. glutinosa*. However, the stable and heritable yellow flower materials found in the present study indicate that there may be a previously unrecognized genetic variation mechanism underlying flower color regulation in *R. glutinosa*. Explaining the metabolic basis and molecular regulation mechanism of this color variation not only enhances our understanding of the genetic diversity in *R. glutinosa*, but also provides the theoretical basis for improvement of color traits. By integrating metabolomics, transcriptomics, and whole-genome resequencing-based variant datasets, the present study systematically dissected the metabolic and molecular mechanisms associated with the flower color variation in *R. glutinosa*, offering important insights for flower color modification and improvement in plants.

Metabolomic analyses revealed that the combined decrease in anthocyanins and accumulation of carotenoids constitutes the core metabolic basis underlying the yellow corolla phenotype in *R. glutinosa*. Cyanidin-based anthocyanins are the predominant pigments responsible for red coloration in plant petals [43,44]. In the present study, it was found that the content of cyanin-3-O-glucoside in YH was significantly lower than that in RH, indicating that its reduction could be a key factor leading to the loss of red pigmentation. Similar observations have been reported in other species, such as the high levels of cyanidin-3-O-glucoside detected in the red sepals of *Cymbidium lowianum* [45]. Comparative analyses of pink and red strawberry petals also showed a significantly higher proportion of cyanidin-type anthocyanins in red flowers [46]. In contrast to the pronounced decline in anthocyanins, the substantial accumulation of lutein in YH provided the primary pigment source for the yellow phenotype. Carotenoids are widely associated with yellow or orange floral coloration, and in species such as *Gentiana lutea*, lutein typically represented the most abundant carotenoid component, consistent with our findings [47]. Taken together, it was speculated that the yellowing in *R. glutinosa* resulted from coordinated changes in both pigment categories: the reduction in cyanidin-3-O-glucoside attenuated the red hue, while the increase in lutein enhanced yellow pigmentation, ultimately generating a stable yellow corolla.

Multiple key enzyme genes in the anthocyanin biosynthetic pathway, CHS, CHI, F3H, DFR, and ANS, are regulated by MYB transcription factors. Members of the S6-subgroup of R2R3-MYB proteins serve as core activators of anthocyanin biosynthesis, and their regulatory roles have been extensively demonstrated. Heterologous expression of the *FaMYB1* of the S6-subgroup from strawberry promoted anthocyanin accumulation in tobacco [48]. Overexpression of *AcMYB75* of the S6-subgroup from kiwifruit significantly enhanced anthocyanin deposition in the leaves of *Arabidopsis* plants [49]. *AtMYB75*, a representative member of the S6-subgroup of the R2R3-MYB family in *Arabidopsis*, activated the expression of *DFR* and *ANS* to regulate anthocyanin biosynthesis [50,51,52]. In the present study, *gene-DH2020_040854*, *gene-DH2020_015992*, and *gene-DH2020_037690* were downregulated in YH, suggesting that weakened MYB-mediated transcriptional activation may constitute a key node leading to the repression of anthocyanin biosynthesis. The biological function of *gene-DH2020_015992* has been reported and designated as *RgMYB41*, which can bind to the *ANS* promoter and activate its transcription in *R. glutinosa*. Homologous and heterologous overexpression of *RgMYB41* significantly enhanced anthocyanin accumulation in transgenic plants [15]. Specifically, overexpression of *RgMYB41* increased the pigmentation of tobacco leaves and floral organs, accompanied by coordinated upregulation of anthocyanin pathway enzyme genes [15]. Similarly, overexpression of *RgMYB41* increased anthocyanin content and simultaneously upregulated *CHS*, *CHI*, *F3H*, *F3′H*, *DFR*, and *ANS* in seedlings, leaves, and roots in *R. glutinosa* [15]. Collectively, these lines of evidence indicated that *gene-DH2020_015992* (RgMYB41) functions as a central regulator of anthocyanin biosynthesis in *R. glutinosa*. It was speculated that diminished MYB-mediated transcriptional activation of anthocyanin biosynthetic genes leads to a global suppression of this pathway, which is consistent with the observed drastic reduction in anthocyanins. This provided a molecular explanation for the floral color variation in *R. glutinosa*.

Transcriptomic analysis identified a total of 18,005 DEGs, indicating extensive transcriptional reprogramming in the yellow-flowered variant rather than changes restricted to the anthocyanin biosynthetic pathway. Such large-scale expression alterations suggest that flower color variation was accompanied by broad metabolic and regulatory adjustments. However, it should be noted that DEG-based analyses are inherently constrained by statistical thresholds, and key regulatory genes exhibiting subtle yet biologically meaningful expression changes may not always be captured. Gene regulatory networks often exhibit a “butterfly effect,” whereby minor perturbations in upstream regulators can lead to amplified downstream consequences. In this context, genes involved in anthocyanin biosynthesis were considered phenotypic readouts rather than primary causal factors underlying flower color variation. Accordingly, the present study further focused on S6-subgroup R2R3-MYB transcription factors and genome-level sequence variations to uncover potential upstream causes of the observed transcriptional and metabolic changes associated with yellow flower formation.

At the metabolic and transcriptional levels, the present study has systematically characterized the molecular and regulatory changes associated with floral color variation in *R. glutinosa*. However, the fundamental cause of the downregulation of S6-subgroup R2R3-MYB genes in YH remains to be further investigated. Whole-genome resequencing–based variant analysis revealed genetic variants that may directly affect MYB gene expression and protein function. Resequencing data identified multiple SNPs and InDels within the coding regions of S6-subgroup R2R3-MYB genes. In *gene-DH2020_015992*, three SNPs were detected in the CDS, resulting in amino acid substitutions A12S, Q114E, and G255E, all of which may exert structural or functional impacts. In the A12S substitution, serine was replaced by alanine. Serine contains a hydroxyl group capable of hydrogen bonding and serving as a phosphorylation site, and its replacement with alanine could eliminate this hydroxyl group, potentially weakening local hydrogen bonding capacity and abolishing a possible modification site. As A12S lies near the N-terminal region within the R2 domain, it may also disrupt the structural stability of the R2 repeat. The Q114E substitution introduced a negatively charged glutamate residue, substantially altering surface charge distribution. In G255E, glycine, characterized by the absence of a side chain and high conformational flexibility, was replaced by a bulkier, negatively charged glutamate. This substitution may restrict backbone flexibility and perturb local structure, thereby compromising overall protein stability. Structural modeling results were highly consistent with the above inference, and the local RMSD values for A12S and G255E reached 14.482 Å and 70.459 Å, respectively, with the exceptionally high RMSD of G255E indicating substantial structural deviation. In addition, the change in the distance between residues also supported this viewpoint. These three nonsynonymous mutations in the CDS of *gene-DH2020_015992* may exert cumulative effects that reduce protein stability and consequently weaken its transcriptional activation capacity toward downstream anthocyanin biosynthetic genes. By comparison, the effects of N187S and I223V in *gene-DH2020_040854* may have a relatively weak influence, but the H194_S195insY insertion may interfere with the activation domain and therefore still impose functional consequences. Similar SNP-induced alterations in transcription factor activity have been reported in other species. There were multiple SNPs in the MYB gene *OsC1* that influence leaf sheath coloration in *Oryza rufipogon* [53]. It was speculated that three non-synonymous mutations in *gene-DH2020_015992* may be the key genetic factors that lead to the decline of the transcription factor activity, resulting in insufficient transcriptional activation of the anthocyanin biosynthetic pathway and ultimately suppressing anthocyanin production.

In the present study, all three S6-subgroup R2R3-MYB genes were downregulated in the yellow-flowered *R. glutinosa*. Given that aligners such as Hisat2 tolerate a certain proportion of mismatches and that the number of mutations within these CDS regions was limited, these variants were insufficient to cause RNA-seq mapping failure or systematic underestimation of transcript abundance [23]. Therefore, the reduced expression of MYB genes represented a biologically meaningful phenomenon rather than a technical deviation. To explore the mechanisms underlying MYB gene downregulation, we analyzed variants identified from whole-genome resequencing. Notably, most of these variants were located in noncoding regions, including promoters, enhancers, and other cis-regulatory elements. Variations in these regulatory regions are likely to directly affect transcription efficiency and gene expression levels. For instance, SNPs and InDels within promoter sequences can alter transcription factor binding sites or chromatin accessibility, thereby contributing to the downregulation of MYB genes in the yellow-flowered variant. Collectively, the functional impairment of S6-subgroup MYBs in yellow-flowered *R. glutinosa* was likely attributable to synergistic effects from two sources. On the one hand, amino acid substitutions within MYB functional domains, such as the A12S, Q114E, and G255E mutations in *gene-DH2020_015992*, may weaken transcriptional activation. On the other hand, mutations within potential cis-regulatory elements or changes in other regulatory factors may contribute to reduced MYB transcript abundance. These factors together weakened the activation of MYB on the anthocyanin biosynthesis pathway, preventing expression of key structural genes, including *CHS*, *CHI*, *F3H*, *F3′H*, *DFR*, and *ANS*, which eventually led to a sharp drop in anthocyanin content and a stable yellow corolla phenotype. The major contribution of our study was the identification of critical nonsynonymous mutations within MYB genes regulating anthocyanin biosynthesis, as well as the elucidation of their potential structural and functional impacts. These findings provided new insights into the genetic basis of floral color variation in *R. glutinosa*. Future work involving gene editing or complementation assays will be essential to experimentally validate the functional consequences of these mutations and clarify the causal relationship between MYB variation and floral color phenotypes.

## 5. Conclusions

The present study systematically elucidated the metabolic and genetic basis associated with floral color variation in *R. glutinosa*. The yellow-flowered phenotype was associated with reduced anthocyanin accumulation, particularly cyanidin-3-O-glucoside, together with enhanced carotenoid accumulation. In the yellow-flowered materials, the anthocyanin biosynthetic pathway exhibited broadly reduced expression of key structural genes, accompanied by decreased expression of MYB transcription factors involved in this pathway. *Gene-DH2020_015992*, previously reported as a key MYB transcription factor regulating anthocyanin biosynthesis in *R. glutinosa*, has been shown to promote anthocyanin accumulation through activation of ANS gene. In the present study, *gene-DH2020_015992* harbored multiple nonsynonymous mutations that may affect protein structure and transcriptional activity, potentially contributing to impair anthocyanin biosynthesis. Notably, this study was based on a naturally occurring and stably inherited yellow-flowered variant, rather than genetically engineered materials, and integrated genome-wide sequence variation with protein structural analysis to explore the genetic basis of flower color variation. Collectively, these findings provide new insights into the genetic basis of flower color variation in *R. glutinosa* and offer a foundation for future functional validation and molecular breeding studies.

## Data Availability

The raw sequence reads were deposited in the Genome Sequence Archive (GSA) at the China National Center for Bioinformation (CNCB) under the accession numbers CRA032666 and CRA032741.

## Supplementary Materials

The following supporting information can be downloaded at: https://www.mdpi.com/article/10.3390/biom16010095/s1. Figure S1: OPLS-DA analysis of metabolomic profiles in wild-type and yellow-flowered *R. glutinosa*; Figure S2. Transcriptomic PCA and hierarchical clustering analyses of flower tissues in *R. glutinosa*; Figure S3. Quality assessment of SNP and InDel variants identified in the yellow-flowered *R. glutinosa* genome; Table S1. Primer sequences for subcellular localization and transcriptional activation assays; Table S2. Summary of RNA-seq data quality in *R. glutinosa*; Table S3. Summary of RNA-seq read mapping statistics in *R. glutinosa*; Table S4. List of S6-subgroup R2R3-MYB proteins used for phylogenetic analysis. Table S5. Summary of sequencing data quality assessment and genome alignment statistics in *R. glutinosa*; Table S6. Summary of detected SNPs; Table S7. Summary of InDel variants in genome-wide and CDS region; Table S8. Global RMSD values of MYB protein models; Table S9. Local RMSD values of mutated residue regions; Table S10. Distance changes between mutation sites and neighboring residues.

## Abbreviations

The following abbreviations are used in this manuscript:

ANS	anthocyanidin synthase
CHI	chalcone isomerase
CHS	chalcone synthase
CNCB	at the China national center for bioinformation
DAM	differentially accumulated metabolite
DEG	differentially expressed gene
DFR	dihydroflavonol 4-reductase
F3H	flavanone 3-hydroxylase
FC	fold change
FPKM	fragments per kilobase million
GSA	genome sequence archive
GSEA	gene set enrichment analysis
PCA	principal component analysis
QC	quality control
RMSD	root mean square deviation
TPM	transcripts per million
VIP	variable importance in projection
