# Supplementary figures and images for: Sequence Variations in MYB (v-myb Myeloblastosis Viral Oncogene Homolog) Genes Impair Anthocyanin Biosynthesis and Contribute to Yellow Flower Phenotype in Rehmannia glutinosa
Source: Biomolecules. 2026 Jan 7;16(1):95. doi: 10.3390/biom16010095 (PMC12839424; doi:10.3390/biom16010095)

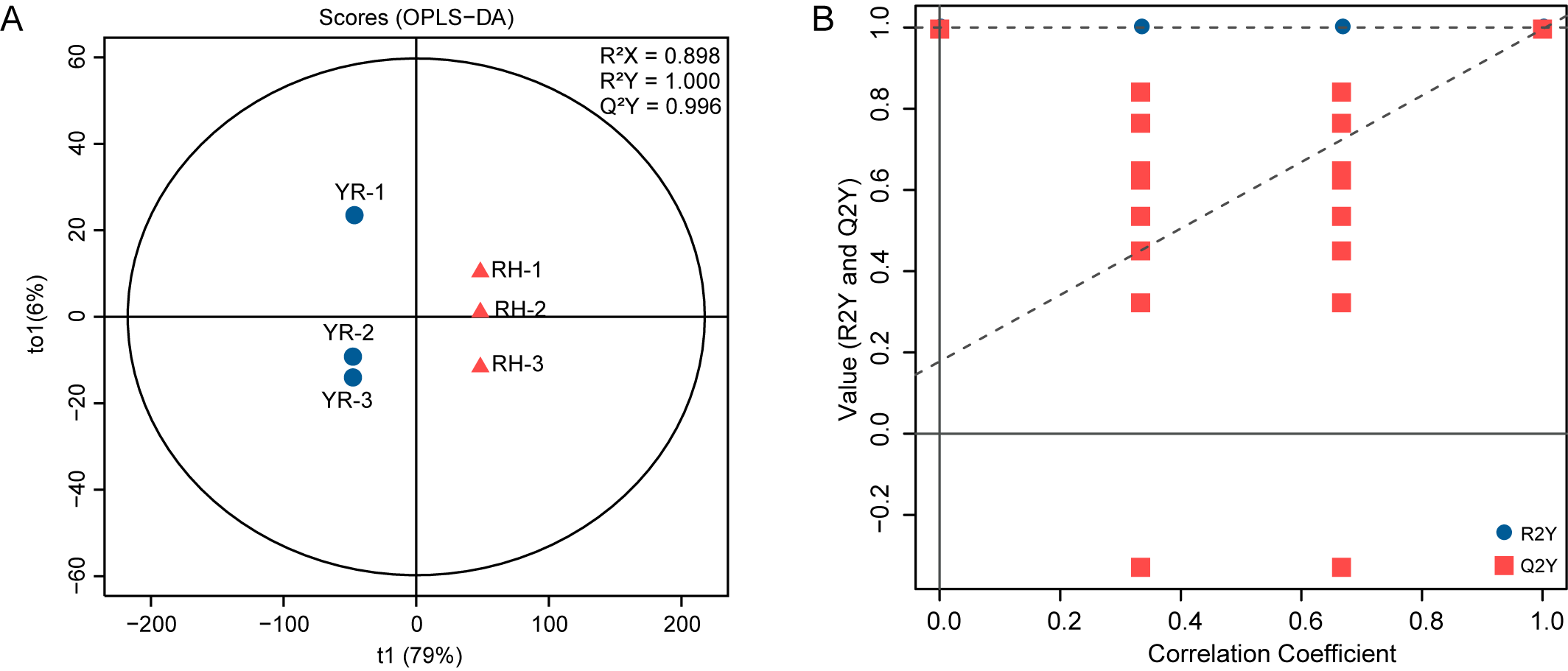

Supplement: Supplementary file 1 [file biomolecules-16-00095-s001.zip › Figure S1.tif]

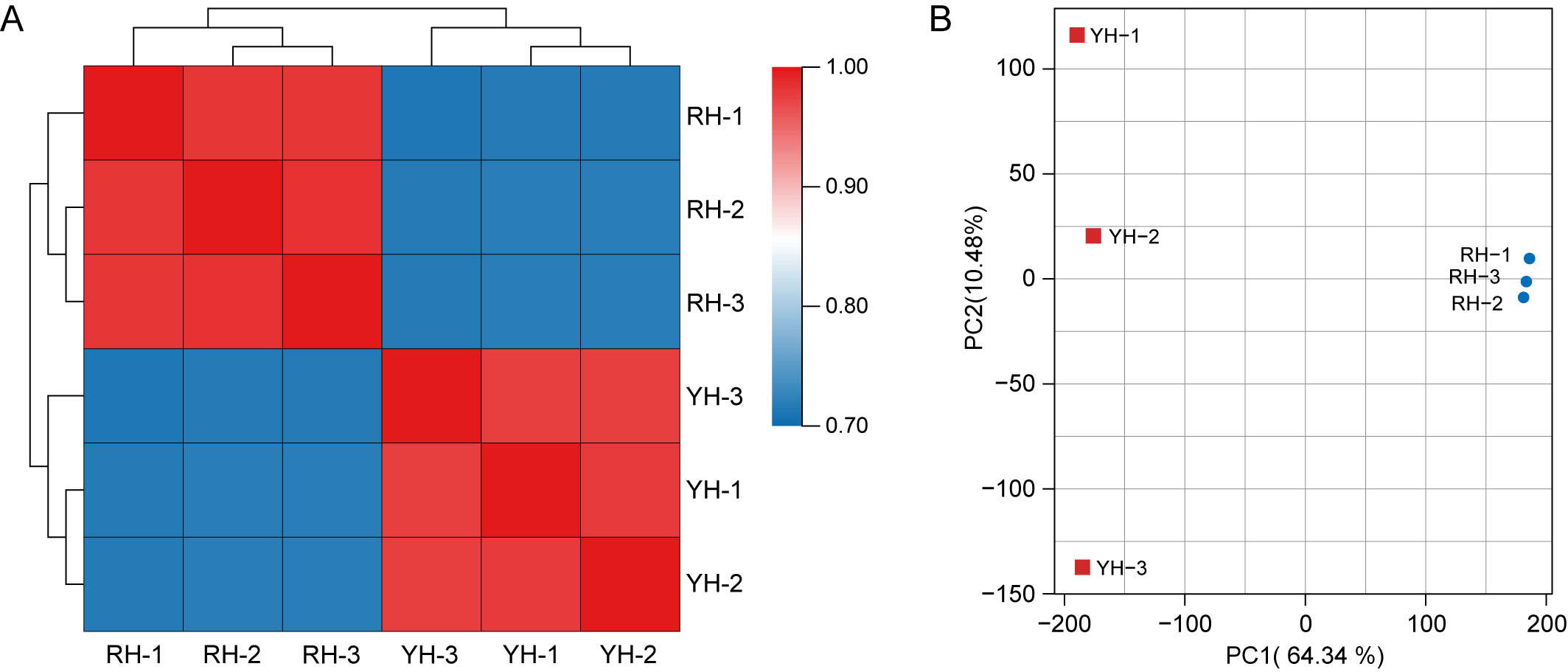

Supplement: Supplementary file 1 [file biomolecules-16-00095-s001.zip › Figure S2.tif]

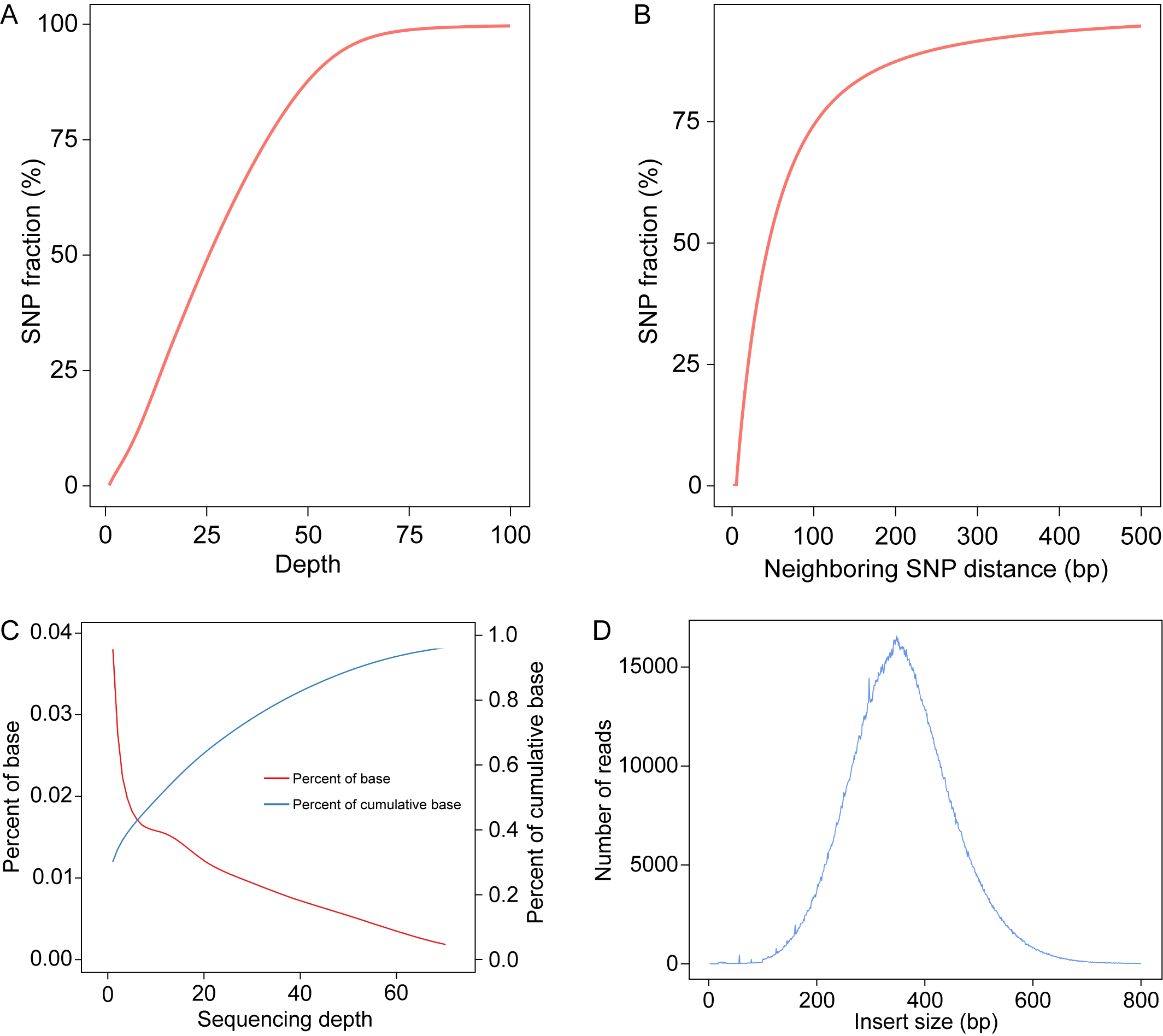

Supplement: Supplementary file 1 [file biomolecules-16-00095-s001.zip › Figure S3.tif]
